# The Treatment of Melancholia

**Published:** 1900-10-20

**Authors:** 


### THE TREATMENT OF MELANCHOLIA.

Lecturing at the Bethlem Royal Hospital on melancholia, Dr. Savage [1] said that, speaking on general principles, in regard to treatment the first thing of all that had to be done was to decide whether the patient was really likely to commit suicide or to attempt it. "If you are sure that a patient is likely to commit suicide," he said, "you have only one real duty, and that is to see that he does not. In nine cases out of ten that means that he has to be placed under restraint by certificate. Patients have to be placed under a certificate more frequently because they are suicidal than from any other cause." The next thing is that, if the patient is refusing food, he should be kept in bed and thoroughly fed. Patients are more easily watched if they are kept in bed than otherwise, but it is well to remember that the choice of a bedroom is an important matter. If melancholics have to be treated in bed they should not be placed at the top of the house for fear of their throwing themselves out of the window. Then there is sleeplessness, and for this drugs may have to be used. Bromides in any case of restlessness and over-excitability are very useful, but food and alcoholic stimulants, given at night, often act as well, or even better. If there has been extreme sleeplessness, morphia is perhaps to be preferred to anything else. If a patient has really been without sleep for several nights, then probably a quarter of a grain of morphia, repeated in a few hours if sleep is not obtained, will be the best thing. The great point is to vary the hypnotics used. The fashionable one now is trional, in 15 gr. to 20 gr. doses. In the Bethlem Hospital sulphonal is used; in other asylums paraldehyde in drachm doses is preferred. It is rather a cardiac stimulant, and therefore has certain advantages, the great disadvantage being that it is utterly distasteful, so that it is not surprising that people who have had two or three doses of paraldehyde say that they have been poisoned. In some cases in which sleeplessness continues, notwithstanding that the general health has improved, general measures may be of service. For instance, a very hot bath the last thing at night, or a hot bath with a certain amount of mustard in it, may give rest very much more readily than anything else, and may thus break the habit of sleeplessness. Both sleeplessness and refusal of food may become mere habits which want breaking. There are certain individuals who get a habit of refusing their food. "I remember going to a private asylum," said Dr. Savage, "and the doctor said, 'Excuse me for a short time; I am going to load the cannon.' I found that the cannon was a canon of the Church of England who was fed regularly three times a day, and that was about the three thousandth time he had been fed by the pump. I think you must break a habit of that kind in the same way that you must break the sleepless habit. A good plan is to put salt in the food and drink, and stand a glass of milk at the side of the patient; the salt makes him thirsty and he drinks the milk."

[1] Clinical Journal, Oct. 3.

